# Modern Hopfield Networks for graph embedding

**DOI:** 10.3389/fdata.2022.1044709

**Published:** 2022-11-17

**Authors:** Yuchen Liang, Dmitry Krotov, Mohammed J. Zaki

**Affiliations:** ^1^Department of Computer Science, Rensselaer Polytechnic Institute, Troy, NY, United States; ^2^MIT-IBM Watson AI Lab, IBM Research, Cambridge, MA, United States

**Keywords:** Modern Hopfield Network, Dense Associative Memory, graph embedding, graph representation learning, node classification, link prediction, graph coarsening

## Abstract

The network embedding task is to represent a node in a network as a low-dimensional vector while incorporating the topological and structural information. Most existing approaches solve this problem by factorizing a proximity matrix, either directly or implicitly. In this work, we introduce a network embedding method from a new perspective, which leverages Modern Hopfield Networks (MHN) for associative learning. Our network learns associations between the content of each node and that node's neighbors. These associations serve as memories in the MHN. The recurrent dynamics of the network make it possible to recover the masked node, given that node's neighbors. Our proposed method is evaluated on different benchmark datasets for downstream tasks such as node classification, link prediction, and graph coarsening. The results show competitive performance compared to the common matrix factorization techniques and deep learning based methods.

## 1. Introduction

The network embedding task is to represent the node of the network as a low-dimensional vector while retaining the topological information (usually reflected by the first-order proximity or second-order proximity). In order to build a good embedding, the model has to extract and store the common topological structure in the network, and use that information to guide the embedding, so that two nodes with similar topological structures have similar encodings. Most existing network embedding methods do matrix factorization explicitly (such as graph Laplacian Eigenmaps factorization; Hofmann and Buhmann, 1995) or implicitly (such as Deepwalk; Perozzi et al., 2014). In this work, we learn the graph structure directly from the data and associate the nodes with the learned structural prototypes stored in the network, which are also used to create node embeddings.

Associative Memories are systems which are closely related to pattern recognition, retrieval and storage. In a typical Associative Memory task a group of stimuli are stored as a multidimensional memory vector. When certain subset of the stimuli is activated, the network recalls the related stimuli stored in the same or related memories. For example, in image datasets, pixel intensities can be associated with label of the image; when a certain part of the image shows up, the network should be able to recall the label. The Hopfield Network (Hopfield, 1982, 1984) is the simplest mathematical implementation of this idea. The information about the dataset is stored as a collection of attractor fixed points (memories) of a recurrent neural network. The input state is iteratively updated so that it moves closer to one of the stored memories after every iteration. The convergence dynamics can be described using the temporal evolution of the state vector over an energy landscape. Classical Hopfield Networks, however, can store and successfully retrieve only a small number of memories, which scales linearly with the number of feature neurons in the network (Crisanti et al., 1986; Torres et al., 2002; Hertz, 2018). Recently, Modern Hopfield Networks (Krotov and Hopfield, 2016), also known as Dense Associative Memories, have been proposed. This new class of models modifies the energy function and the update rule of the original Hopfield Network to include stronger (more sharply peaked around memories) non-linear activation functions. This results in a significant increase in the memory storage capacity making it super-linear in the dimensionality of the feature space. Later studies (Demircigil et al., 2017) extend the interaction term of the Modern Hopfield Network, which leads to exponential storage capacity. Additionally, (Krotov and Hopfield, 2016, 2020; Ramsauer et al., 2020) extend the Hopfield Network to continuous states. It has also been shown that the attention mechanism can be regarded as a special case of Hopfield Network with a certain update rule and energy function (Krotov and Hopfield, 2020; Ramsauer et al., 2020).

In our work, we tackle the network embedding problem from the brand new angle of MHNs, which are recurrent attractor networks that store information about the network in the form of memories (fixed points of the iterative temporal dynamics). This is a completely alternative representation compared to the conventional methods based on feedforward neural networks, e.g., Graph Convolutional Networks (GCNs). Feedforward networks are regression tools that use their parameters to draw sophisticated decision boundaries that separate different classes of nodes. Hopfield Networks, on the other hand, form basins of attraction around “prototypical node classes” that resemble many individual nodes from the training set. These prototypical nodes constitute the memory matrices, which can be learned during training. As such, these matrices are not just arbitrary parameters of the feedforward network, but rather are interpretable descriptors of the attractor states (local minima of the energy function). A good node embedding should benefit from these summarized neighborhood patterns learned from the data. Thus, it is very natural to use these descriptors for constructing node embeddings.

Driven by this idea, our work proposes to view the network embedding task as an Associative Memory problem. The memories of the Modern Hopfield Network are used as trainable parameters that learn to store the topological information of the network. We show how the recurrent dynamics of the Associative Memory network can be used to predict the masked nodes, and help us generate node embedding based on the memories learned from the data. The main contributions of our paper are as follows:

- We design an Associative Memory update rule and its corresponding energy function suitable for the network embedding task.
- We empirically show that the performance of our MHN-based embedding for the node classification, link prediction and graph coarsening downstream tasks is competitive with the commonly used matrix factorization methods and deep learning approaches.

The rest of the paper is organized as follows. We first discuss the related work in Section 2. Our proposed model for node embeddings based on Modern Hopfield Networks is presented in Section 3; this includes the model description, training and complexity. Next, in Section 4 we present a detailed experimental evaluation of our model on two main downstream applications: node classification and link prediction, and graph coarsening. For the latter, we further evaluate our model on the graph classification and block structure identification tasks. We summarize our conclusions in Section 5. Finally, the Appendix includes details of our energy function, as well as an ablation study of our model for the node and link prediction tasks, and further analysis on the graph classification task.

## 2. Related work

### 2.1. Graph or network embedding

There are mainly two types of approaches for the homogeneous network embedding task: matrix factorization based approaches and deep learning based approaches.

The matrix factorization based approaches factorize some matrix which reflects the topological information of the network. There are mainly two different directions: one is to factorize the graph Laplacian Eigenmaps (Anderson Jr and Morley, 1985; Hofmann and Buhmann, 1995), and the other is to factorize the node proximity matrix (Golub and Reinsch, 1971). For a given network *G* = (*V, E*) with *m* nodes, graph Laplacian Eigenmaps factorization lets similar node embeddings have similar values; the high similarity nodes with very different embeddings are heavily penalized. Different approaches utilize different ways to create the node similarity matrix. For instance, Hofmann and Buhmann (1995) use Euclidean distance between the feature vectors, Anderson Jr and Morley (1985) and He and Niyogi (2004) construct the *k*-nearest neighbor graph to enhance the local connections, and Jiang et al. (2016) use an anchor graph, which is shown to be effective at preserving the local projection. For node proximity matrix factorization, the goal is to minimize the loss of approximating the proximity matrix directly. Different methods have different ways of constructing the proximity matrix and different factorization techniques. GraRep (Cao et al., 2015) leverages *k*-hop transfer information to construct the proximity matrix and uses Singular Value Decomposition for the factorization. Ou et al. (2016) use different similarity metrics for quantifying the proximity matrix, and use generalized SVD to speed up the computation. Yang et al. (2015) uses low-rank matrix factorization over the Pointwise Mutual Information matrix and add context information during the factorization. Unify LINE (Tang et al., 2015b; Qiu et al., 2018) and PTE (Tang et al., 2015a), which do implicit matrix factorization for different proximity matrices, and propose different proximity matrices for small and large window sizes. Recently, Zhu et al. (2021) proposed a unified architecture for the general embedding process.

For the deep learning based approaches, one direction of research is related to random walks, where the whole network can be represented by a set of random walks starting from random nodes in the network. A node's neighbor information can be reflected by the neighbor information in the random walk sequence. Then we can get node embeddings by embedding the random walk sequences. Inspired by word2vec (Mikolov et al., 2013a) in the natural language processing area, DeepWalk (Perozzi et al., 2014) utilizes the SkipGram model to maximize the probability of seeing the neighboring nodes in the node sequence, conditioned on the node embedding itself. Hierarchical softmax and negative sampling (Mikolov et al., 2013b) is used to increase the model efficiency. Many subsequent studies try to improve the graph diffusion process in order to better model the representation of the network. For example, node2vec (Grover and Leskovec, 2016) uses both the breadth first search (BFS) and depth first research (DFS) to control the breath and depth of the exploration. BFS helps to express the neighbor information and DFS helps to reflect the global information of the network. Aside from random walk based approaches, there are other deep learning based methods such as SDNE (Wang et al., 2016) and SAE (Tian et al., 2014) based on autoencoders, where SDNE exploits the first-order and second-order proximity jointly to preserve the network structure and SAE is a sparse autoencoder network.

It is also worth noting that there are network embedding methods that can scale to larger graphs by utilizing hierarchical or coarsening methods to speed up different matrix factorization or deep learning methods (Chen et al., 2018; Liang et al., 2021). Our goal is somewhat different—we use a new embedding model based on Associative Memories, and further we show that those embeddings capture relevant structural information that can also be used to coarsen a graph, among several other downstream tasks.

### 2.2. Associative memories

Associative Memories are systems which are closely related to pattern recognition, retrieval and storage. The Hopfield Network (Hopfield, 1982, 1984) is the simplest mathematical implementation of this idea. The information about the dataset is stored as a collection of attractor fixed points (memories) of a recurrent neural network. However, the memory storage capacity for the traditional Hopfield network is small; in a *d*-dimensional space, the network can only store 0.138*d* memories (Hopfield, 1982; Amit et al., 1985; Hertz, 2018). Modern Hopfield Networks (Krotov and Hopfield, 2016), also known as Dense Associative Memories, modify the energy function and the update rule of the original Hopfield Network to include stronger (higher-order in terms of interactions) non-linear activation functions. This results in a significant increase in the memory storage capacity making it super-linear in the dimensionality of the feature space. For certain choices of the activation functions, even an exponential storage capacity is possible (Demircigil et al., 2017). Modern Hopfield Networks with continuous states have been formulated in a series of papers (Krotov and Hopfield, 2016, 2020; Ramsauer et al., 2020; Krotov, 2021). It has also been shown that the attention mechanism can be regarded as a special case of the MHN with certain choice of the activation function (softmax) for the hidden neurons (Krotov and Hopfield, 2020; Ramsauer et al., 2020).

## 3. Modern Hopfield Networks for node embeddings

Consider a network *G* = (*V, E*). Each node *v*∈*V* in the network can be represented by its context vector, denoted by *v*_context_. The context is one or more hops neighbors around *v*, resulting in a *m* = |*V*| dimensional binary context vector $v_{\text{context}}\in {\left\{0,1\right\}}^{m}$. For node classification tasks, the desired target node embedding can be represented as the one-hot encoding for the node itself, which serves as the ground truth when computing the loss. The target node embedding is also an *m*-dimensional binary vector.

### 3.1. Iterative updating rule

**Figure 1:**
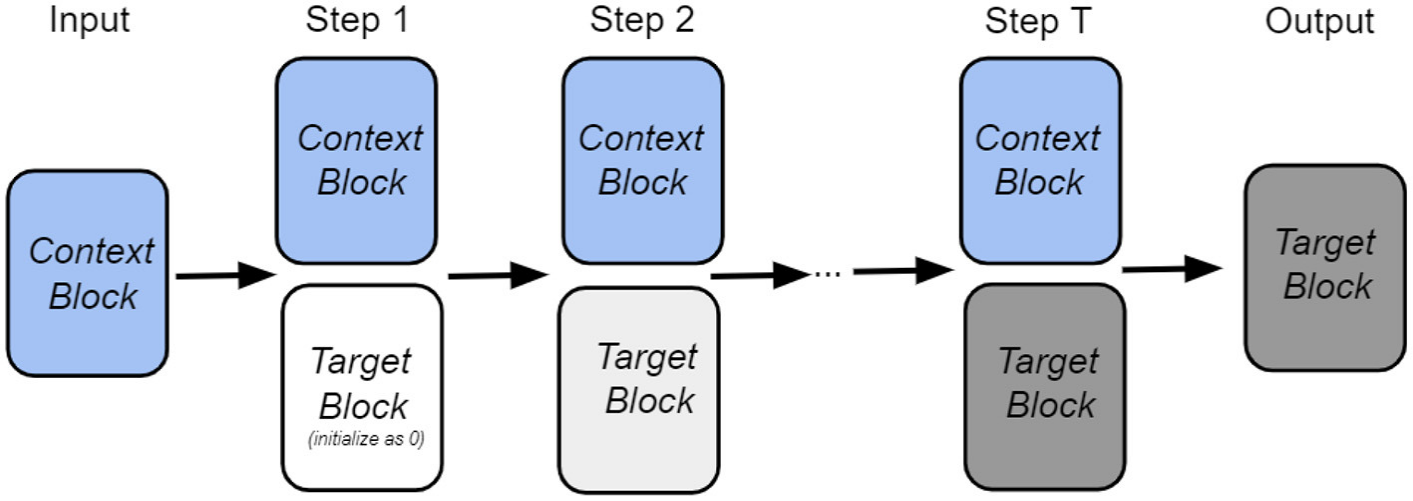
The overall architecture of our iterative node retrieval process.

Intuitively, our goal is to retrieve the target node from the memory with the help of the input context information and information from the previous step. The overall architecture of our retrieval process is shown in Figure 1. As shown in the figure, the state of the network is described by two vectors: *v*_context_, which encodes the neighbors of a given node, and $v_{\text{target}}^{\left(t\right)}$, which encodes the target node at time step *t*. Each block has its own set of trainable weights: Ψ_context_ and Φ_target_, which serve as memories in the Associative Memory network. At the initial moment of time, the context information is presented to the network, and the target node is masked. The Associative Memory dynamics then predict the target node after several recurrent iterations. During the iterative retrieval process, the context vector *v*_context_ is fixed (shown as context block in the figure) for each step, while the target vector evolves with time.

During the retrieval stage the update rule for our network is given by


(1) $$\begin{array}{l} D_{\text{sim}}=f\left(\beta_{1}\Phi_{\text{target}}v_{\text{target}}^{\left(t\right)}+\beta_{2}{\text{Ψ}}_{\text{context}}v_{\text{context}}\right) \end{array}$$

(2) $$\begin{array}{l} v_{\text{update}}^{\left(t\right)}=\Phi_{\text{target}}^{T}D_{\text{sim}} \end{array}$$

(3) $$\begin{array}{l} v_{\text{target}}^{\left(t+1\right)}=v_{\text{target}}^{\left(t\right)}+\alpha \left(v_{\text{update}}^{\left(t\right)}-v_{\text{target}}^{\left(t\right)}\right) \end{array}$$

where *v*_context_ is the input context encoding, $v_{\text{target}}^{\left(t\right)}$ is the target encoding at the step *t*. At the beginning of the retrieval dynamics $v_{\text{target}}^{\left(0\right)}$ is initialized as a vector of zeros. That is, our model can be considered as a masked target node prediction model, such that we predict the target purely from the context vector *via* the system dynamics. The matrices $\Phi_{\text{target}}\in R^{K\times m}$ and ${\text{Ψ}}_{\text{context}}\in R^{K\times m}$ are memories stored in the network for target and context blocks, respectively. We store *K* context memory patterns and *K* target memory patterns, where each memory pattern is an *m*-dimensional vector. Both Ψ_context_ and Φ_target_ are learnable parameters, *f* is the softmax function, and parameters β_1_ and β_2_ control the temperature of the softmax. *D*_sim_ is the similarity between the current pattern and all the patterns stored in the network (considering both context block and target block), $v_{\text{update}}^{\left(t\right)}$ is the readout from the memory for the target block. The constant α is the update rate for each step. It is a hyperparameter of our model, along with β_1_ and β_2_.

**Figure 2:**
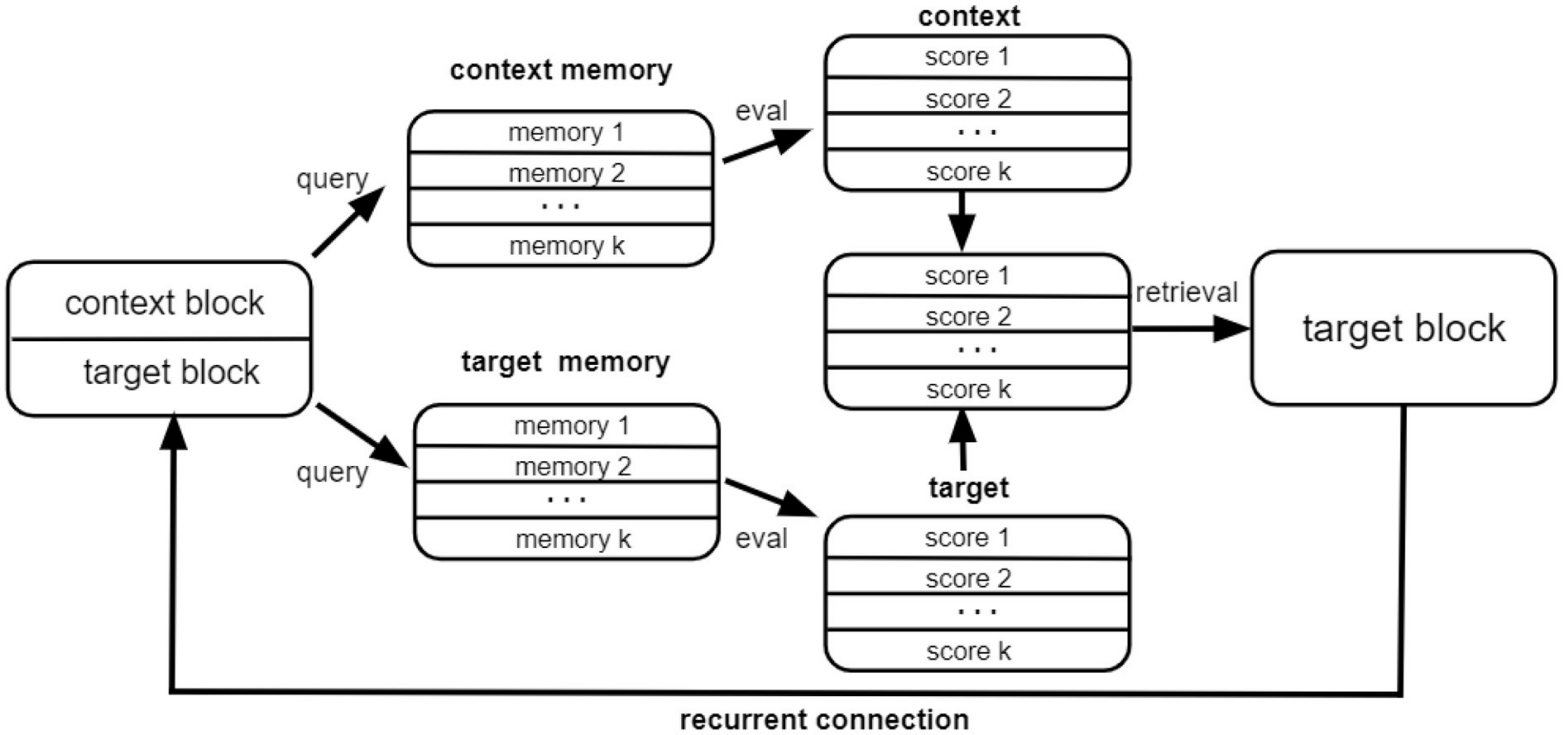
The architecture of our network within each step.

Intuitively, at every step our approach tries to retrieve the correct target information $v_{\text{update}}^{\left(t+1\right)}$ from the memory with the help of the context information *v*_context_ as well as the target information from the previous step $v_{\text{target}}^{\left(t\right)}$. The target block state is gradually updated until it becomes stable. The network architecture within each step is illustrated in Figure 2. As illustrated, for each retrieval step, the input to the module consists of the context block encoding the node feature information as well as the target block, which is equal to the state of the network from the previous iteration step. The context block and the target block query the context memory and the target memory, respectively. The score proportional to the overlap of the state vector and the memory matrix is used to retrieve the memory attentive target block, which serves as the output of the module. The whole process can be iterated for several time steps.

#### 3.1.1. Stored memories as energy minima

The above network architecture is a special kind of the continuous state and continuous time Modern Hopfield Network (Krotov and Hopfield, 2020). It can be shown that the network's updating process is minimizing the following Lyapunov energy function


$$\begin{array}{c} E=\frac{1}{2}\sum_{i=1}^{m}{\left(v_{\text{target}}\right)}_{i}^{2}-\log[\sum_{\mu =1}^{K}\exp(\sum_{i=1}^{m}{\left(\Phi_{\text{target}}\right)}_{\mu i}{\left(v_{\text{target}}\right)}_{i}\\ \;+\epsilon_{\mu })] \end{array}$$

where (_*v*_target_)*i*_ stands for the *i*-th element of the target vector state *v*_target_, (_Φ_target_)μ*i*_ stands for the *i*-th element for the μ-th target memory, and $\epsilon_{\mu }=\overset{m}{\underset{i=1}{\sum }}{\left({\text{Ψ}}_{\text{context}}\right)}_{\mu i}{\left(v_{\text{context}}\right)}_{i}$, where (_*v*_context_)*i*_ stands for the *i*-th element for the input context vector, and (_Ψ_context_)μ*i*_ stands for the *i*-th element for the μ-th context memory. The details of energy function derivation are shown in Appendix. The energy monotonically decreases as the dynamics progress. Eventually, the state of the network will converge to the local minimum corresponding to one of the stored memory patterns.

### 3.2. Training and embedding generation

In the training phase, we collect the encoding of the target block $v_{\text{target}}^{\left(T\right)}$ after *T* steps of the iterative dynamics when the retrieval is stable, and then compute the cross entropy loss between this target state and the actual encoding for the target node (which is represented as a one hot encoded vector). This loss function is used for training the memory matrices Ψ_context_ and Φ_target_ using the backpropagation algorithm.

In the embedding generation phase after the training is complete, the *K*-dimensional embedding for each node can be computed using the following equation


$$\text{node embedding}={\text{Ψ}}_{\text{context}}v_{\text{context}}$$

where $v_{\text{context}}\in {\mathbb{R}}^{m}$ is the context encoding for that node, and ${\text{Ψ}}_{\text{context}}\in R^{K\times m}$ is the memory matrix for the context block, which has already been learned during the training phase. Intuitively, each element of the final embedding indicates a similarity score between the input context vector and specific memories stored in the Dense Associative Memory network.

### 3.3. Complexity analysis

The most expensive part of our approach is the matrix multiplication $\Phi_{\text{target}}v_{\text{target}}^{\left(t\right)}$, where $\Phi_{\text{target}}\in R^{K\times m}$ and $v_{\text{target}}^{\left(t\right)}\in R^{m\times B}$ (*K* is the number of memories, *m* is the number of nodes and *B* is the batch size). The complexity for this matrix multiplication is O(KBm). For each batch of data, we iterate *T* steps. Thus, the time complexity per epoch is $O\left(KTm^{2}\right)$. Since both *K* and *T* are constant, the total time complexity is $O\left(m^{2}\right)$, which is the same as other baseline methods such as LINE (Tang et al., 2015b) and SDNE (Wang et al., 2016).

## 4. Experiments

In this section, we empirically evaluate the performance of our proposed network embedding model in node classification and link prediction downstream tasks on commonly used benchmarks. Besides, we further demonstrate that our embedding can be very useful for the graph coarsening task.

### 4.1. Node classification and link prediction

For both of these two downstream tasks, we first generate node embeddings in an unsupervised way by using different baseline methods. Then we use the same classifier on top of the learned embeddings for the evaluation. Thus, the downstream task accuracy reflects the quality of the embeddings.

#### 4.1.1. Datasets

We first learn unsupervised node features purely from network structure and then report the performance of our embeddings for multi-label classification and link prediction downstream task on three datasets: BlogCatalog (Reza and Huan, 2009), Protein-Protein Interactions (Oughtred et al., 2019), and Wikipedia (Mahoney, 2011; Grover and Leskovec, 2016). BlogCatalog is a network of social relationships reflected by a blog user, and the label is indicated by the categories of the blogs. Protein-Protein Interactions is a network which indicates the interactions of proteins that are experimentally documented in humans, and the label indicates the biological state. Wikipedia is a word co-occurrence network for the first 10^9^ bytes of the English Wikipedia dump. There exists an edge between words co-occurring in a 2-length window. The statistics of the networks and the number of labels/categories for the nodes are summarized in Table 1.

**Table 1:** Graph statistics for the datasets.

	**Blogcatalog**	**PPI**	**Wikipedia**
|*V*|	10,312	3,890	4,777
|*E*|	333,983	76,584	184,812
Categories	39	50	40

#### 4.1.2. Baseline methods and metrics

We compare our embedding approach against DeepWalk (Perozzi et al., 2014), node2vec (Grover and Leskovec, 2016), LINE (Tang et al., 2015b), and PhUSION (Zhu et al., 2021), which are commonly used approaches for learning the latent node representations for a network. DeepWalk first represents the network by a set of random walks starting from random nodes in the graph, so that a node's neighbor information can be reflected by the neighbor information in the random walk sequence. The node embedding is obtained by embedding the random walk sequences using the SkipGram model (Mikolov et al., 2013a). Node2vec is a modification of DeepWalk with a small difference in random walks, using two parameters to control the breadth and depth of the exploration. LINE optimizes a carefully designed objective function that preserves both the local and global network structures. PhUSION proposes a unified architecture of the general embedding process, which consists of node proximity calculation, nonlinear transformation function, and embedding functions.

For the node classification downstream task, we follow the procedure of previous methods, training a one-vs.-rest logistic regression model *via* the LibLinear library (Fan et al., 2008) on top of all the embeddings for the classification task. We report the micro-f1 and macro-f1 scores based on the average performance of 20 runs, and we also report the standard deviation across those runs. In our experiments, the train/test split for evaluation is 9:1. For our embedding model, we use 2,000 memories across all datasets. For baseline models we run the publicly available codes using the default settings. All the parameters are learned by the backpropagation algorithm.

For the link prediction downstream task, we randomly sample 500 positive and negative node pairs, respectively, for each dataset. The task is to predict whether or not there is a connection between the node pairs based on their nodes' embeddings. The probability of an edge between nodes *i* and *j* is given by $\sigma \left(h_{i}^{T}h_{j}\right)$, where σ is the logistic sigmoid function. The vectors *h*_*i*_ and *h*_*j*_ are the node embeddings. We plot the Receiver Operating Characteristic (ROC) curve, and also report the Area Under the Curve (AUC) scores for all the embedding methods. Also, we include a heuristic method using Jaccard's Coefficient for comparison. The Jaccard's Coefficient is defined as $\frac{\left|N\left(u\right)\cap N\left(v\right)\right|}{\left|N\left(u\right)\cup N\left(v\right)\right|}$ for a given node pair (*u, v*) with the immediate neighbor sets *N*(*u*) and *N*(*v*), respectively. We use python scikit-learn built-in function for the ROC curve drawing and AUC score computation.

For our graph node embedding generation, Adam optimizer and weight decay is used during training, and learning rate is initialized as 0.01. Parameter β_1_ is 1, β_2_ is 0.5, and update rate α is 0.2. The hyperparameters are selected based on the validation set.

#### 4.1.3. Empirical evaluation

##### 4.1.3.1. Node classification

Table 2 summarizes the results for graph node embeddings generated by different methods on the downstream node classification task. Our MHN based approach outperforms DeepWalk, node2vec, LINE, and PhUSION on two out of three datasets both for micro- and macro-f1 scores. On BlogCatalog our method loses to DeepWalk, but still performs better than other methods. Associative Memory network works particularly well on the Wikipedia dataset resulting in more than 7% improvement over DeepWalk and node2vec, and over 6% improvement over LINE for the micro-f1 score.

**Table 2:** Average micro-f1/macro-f1 scores for multi-label classification task.

**Dataset**	**Ours**	**DeepWalk**	**node2vec**	**LINE**	**PhUSION**
Blogcatalog	40.26^±0.53^/24.23^±0.97^	**42.76^±1.28^**/**28.48^±1.36^**	38.24^±1.23^/21.61^±1.42^	37.19^±1.15^/20.59^±1.35^	18.03^±0.72^/3.58^±0.22^
PPI	**26.11^±1.48^**/**21.29^±1.36^**	23.39^±1.29^/19.19^±1.51^	22.19^±1.78^/18.63^±2.09^	21.55^±1.56^/17.75^±1.88^	11.17^±0.14^/5.25^±0.71^
Wikipedia	**57.62^±0.98^**/**13.84^±0.73^**	50.56^±1.38^/10.19^±1.11^	50.13^±1.68^/9.73^±0.85^	51.21^±1.81^/10.31^±1.02^	43.82^±1.71^/5.51^±0.59^

We use 7 steps for unfolding in time during the memory retrieval pass. Our model has 2,000 hidden units (memories) for each block.

Bold value stands for the best performance for each dataset.

##### 4.1.3.2. Link prediction

**Figure 3:**
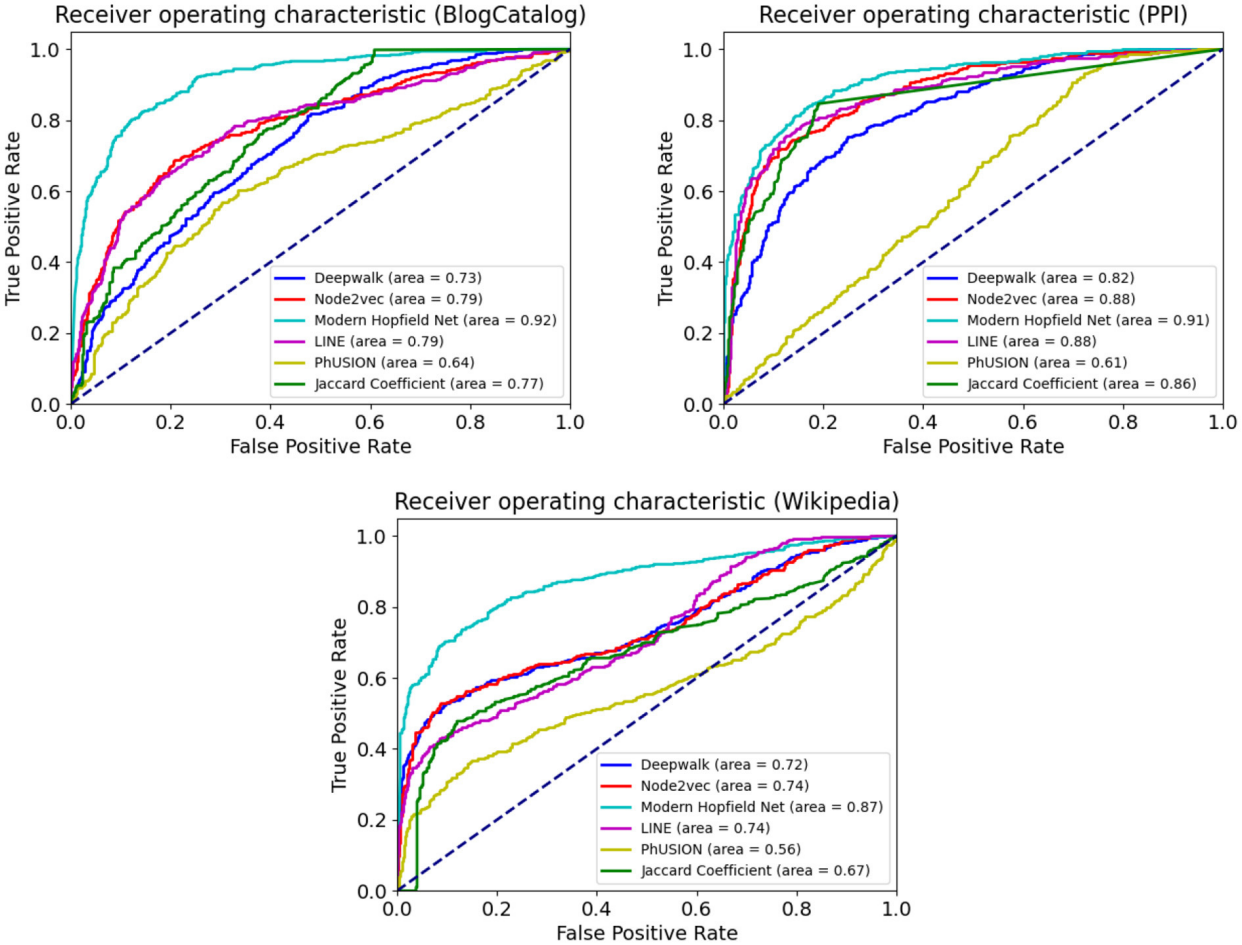
The Receiver Operating Characteristic (ROC) curve for the link prediction task on BlogCatalog, PPI, and Wikipedia. Comparison with popular baseline methods.

Table 3 and Figure 3 summarize our results for the downstream link prediction task. For every pair of nodes, the dot product of their embedding vectors is passed through a sigmoid function and thresholded at a certain value. Scores above the threshold are predicted as links, and below the threshold as absence of links.

**Table 3:** Area under curve (AUC) scores for link prediction.

**Dataset**	**Ours**	**DeepWalk**	**node2vec**	**LINE**	**Jaccard's coefficient**	**PhUSION**
BlogCatalog	**0.92** (16%)	0.73	0.79	0.79	0.77	0.64
PPI	**0.91** (3%)	0.82	0.88	0.88	0.86	0.61
Wikipedia	**0.87** (17%)	0.74	0.74	0.72	0.67	0.56

Comparison with popular baselines. The number in parenthesis shows the performance gain when compared with the second best baseline.

Bold value stands for the best performance for each dataset.

The ROC curves are obtained as the discrimination threshold is varied. Our Associative Memory based method does extremely well on the link prediction task across all the benchmark datasets. Such a strong performance is expected from the conceptual computational design of our network. On the one hand, nodes with similar neighborhood structure tend to have a link connecting them. On the other hand, nodes with similar neighborhood structure will be more likely attracted (in the course of the Hopfield dynamics) by the same group of memories. Thus, the core computational strategy of our model is particularly well-suited for this task.

### 4.2. Graph coarsening

As large graph datasets become more common in practical machine learning applications, computational efficiency becomes a new challenge we need to cope with, since most existing graph based methods struggle to deal with graphs with hundreds and thousands of nodes. One way to address this issue is to sample the subgraph to train the model. Another way to address this problem is to do graph coarsening, which aims to produce a simpler graph with fewer nodes and edges while preserving important properties of the original graph. Graph coarsening is particularly useful for those graph algorithms that cannot be trained in a batch fashion. Also, recent studies (Chen et al., 2018; He et al., 2021; Liang et al., 2021) show that the coarsened graph can also facilitate graph embedding. In this section, we demonstrate that our embedding methods can be very useful for the graph coarsening task.

#### 4.2.1. Graph coarsening with node merging

Following the unsupervised learning algorithm described in the previous sections, we are able to obtain the embeddings for each node in the input graph. Here we propose a hierarchical way to do the graph coarsening with node merging using those learned node embeddings. The overall approach is shown in Algorithm 1.

**Algorithm 1:** Graph coarsening with node merging.


**Input**: Graph *G* = (*V, E, W*), target size of
coarsened graph *n*, and pairwise node
similarity matrix *S*
**Output**: Graph *G*_*c*_ = (*V*_*c*_, *E*_*c*_, *W*_*c*_)
*G*_*c*_ = *G*
*S*_*c*_ = *S*
**while** *n* < |*V*_*c*_| **do**
*p, q* = maximal similarity finder(*S*_*c*_)
*S*_*c*_ = similarity merger(*S*_*c*_, *p, q*)
*G*_*c*_ = node merger(*G*_*c*_, *p, q*)
**end while**

The pairwise node similarity matrix *S*∈*R*^*m*×*m*^ can be obtained from the node embeddings (*m* is the number of nodes), and is defined as


$$S\left(i,j\right)=\text{cosine similarity}\left(v_{\text{embed}}^{i},v_{\text{embed}}^{j}\right)$$

where $v_{\text{embed}}^{i}$ and $v_{\text{embed}}^{j}$ are the embeddings for nodes *i* and *j*, respectively. We use this similarity matrix to find the most similar pair of nodes to merge at each step.

Starting from the original graph as initial state (where each node is in its own group), at every step, we pick two groups with the largest similarity (*p* and *q*) to merge (defined as maximal similarity finder(*S*) in Algorithm 1). The similarity merger is defined as follows: the similarity between the newly merged supernode and the rest of the group is defined as the largest similarity between its group members and the rest of the group (also known as complete link), while the similarity between other groups remain the same as at the previous step. Next, the two groups of nodes are contracted and the weight matrix *W*_*c*_ is adjusted accordingly [defined as node merger (*G, p, q*) in Algorithm 1]. The weight matrix adjustment is usually considered to be the weight accumulation across the group, defined as:


$$W\left(p,q\right)=\underset{v_{i}\in S_{p},v_{j}\in S_{q}}{\sum }W\left(i,j\right)$$

Thus, the weight between the newly created supernode *s* and any other group *i* can be obtained by


W(s,i)=W(p,i)+W(q,i)

while the weights between other groups remains the same as at the previous step. For each loop, the whole coarsening procedure will reduce the number of nodes of the graph by one (at each step two groups merge into one, so the number of groups/nodes decreases by one), and the coarsening process will continue until the target graph size is met. Intuitively, the algorithm iteratively merges the nodes which have similar neighborhood structure into the same group.

We use two tasks to show the effectiveness of graph coarsening based on our embeddings. For all the experiments, we use the number of memories equal to the target coarsened graph size (thus, it varies for different input graphs if the target coarsening size is a fixed ratio of the original size, e.g., one fifth), so as to balance the number of memories used for different graph sizes.

#### 4.2.2. Graph classification

Graph classification is a commonly used task for graph related applications, and the goal is to identify the graph label given the relevant graph information. There are many existing graph classifiers. However, some classifiers [such as Bruna et al., 2013; Henaff et al., 2015, which require eigenvalue decomposition resulting in *O*(*m*^3^) complexity] are not scalable for large graphs, such as those commonly used in social networks studies and computational biology. Graph coarsening can be used to simplify the complicated graph structure without losing valuable information, thus helping the classifier to work efficiently on large graph datasets.

##### 4.2.2.1. Datasets

We evaluate our methods on commonly used graph classification benchmarks and report the classification accuracy on the following datasets: MUTAG (Debnath et al., 1991), ENZYMES (Borgwardt et al., 2005), NCI109 (Wale et al., 2008), PROTEINS (Dobson and Doig, 2003), PTC_MR (Morris et al., 2020), TUMBLR (Morris et al., 2020), OHSU (Morris et al., 2020), and REDDIT-BINARY (Rossi and Ahmed, 2015). Some statistics of the graphs are summarized in Table 4.

**Table 4:** Graph statistics for all the considered datasets.

	**MUTAG**	**ENZYMES**	**NCI109**	**PROTEINS**	**PTC_MR**	**TUMBLR**	**OHSU**	**REDDIT-BINARY**
*N*	188	600	4,127	1,113	344	373	79	2,000
Avg. nodes	17.93	32.63	29.68	39.06	14.29	53.11	82.01	429.63
Avg. edges	19.79	62.14	32.13	72.82	14.69	199.78	199.66	497.75
Categories	2	6	2	2	2	2	2	2

##### 4.2.2.2. Baseline methods and metrics

In this section we explain the baseline methods that we compare with our proposed technique as well as the metrics for comparison. We compare our graph coarsening methods against Edge Matching (EM) (Dhillon et al., 2007), Local Variation (LV) (Loukas, 2019), METIS (Karypis and Kumar, 1998), Spectral Clustering (SC) (Von Luxburg, 2007), HARP (Chen et al., 2018), Multilevel Graph Coarsening (MGC), and Spectral Graph Coarsening (SGC) (Jin et al., 2020), which are commonly used approaches for simplifying the network architecture.

- Edge Matching (EM) gradually merges pairs of nodes with the most heavy weight, with the weight calculated as *W*(*i, j*)/*max*{*d*(*i*), *d*(*j*)}, where *d*(*i*) stands for the degree of vertex *i*. The merging procedure is as follows: starting with all the nodes as unmarked, at each step, visit each node in random order, and for each node, if it's unmarked, merge it together with the neighbor which has the most heavy weight, and mark these two nodes. If all the neighbors of a node are marked, then mark it without any merging. Once all the nodes are marked, one step is finished. This process will iterate until the coarsened graph size is met.
- Local Variation (LV) algorithm starts from a candidate family *F*_*l*_ = {*C*_1_, *C*_2_, ..., *C*_*n*_} where each candidate contraction set *C*_*i*_ is a subset of the neighborhood of a vertex. The algorithm selects a valid graph partition from the candidate family (i.e., non-overlapping while covering all the vertices). The selection is designed to minimize the local variation cost, which captures the maximal variation of all signals from an appropriate subspace.
- METIS is a standard graph partitioning algorithm, which transforms the original graph sequentially to smaller graphs, partitions the smaller graph and then projects the partition back to the original graph while refining using a greedy algorithm at each step.
- Spectral Clustering (SC) selects the eigenvectors corresponding to the top-*k* eigenvalues, and then does the standard K-means algorithm on top of the subspace spanned by the top-*k* eigenvectors.
- MGC gradually merges pairs of nodes with the closest distance. The distance is calculated as follows: $\left|\frac{W\left(i\right)}{d\left(i\right)}-\frac{W\left(j\right)}{d\left(j\right)}\right|$. The algorithm merges one pair of nodes at each time and the process will iterate until the coarsened graph size is met.
- SGC slightly modifies the Spectral Clustering; instead of selecting the eigenvectors corresponding to the top-*k* eigenvalues, they select eigenvectors based on the two ends of the eigenvalues range (the first *p* and the last *k*−*p*).
- HARP is a novel hierarchical way to learn node representation that works by finding a smaller graph to approximate the global graph architecture. We use HARP to get the node representations, and use our merging algorithm for the coarsening steps.
- Aside from the above commonly used graph coarsening methods, we include another baseline which uses graph convolutional neural networks (Kipf and Welling, 2016) to get the node representations, and uses the same node merging algorithm at the coarsening step. We will call it GCN for the rest of the paper.

For all the methods, we coarsen the graph until *n* = *m*/5, i.e., the number of nodes in the coarsened graph is one fifth compared with original graph. Following previous work (Jin et al., 2020), we use the same graph classifier [Network Laplacian Spectral Descriptor (NetLSD) (Tsitsulin et al., 2018) combined with a 1-NN classifier]. We report the classification accuracy for the coarsened graph with different coarsening methods, and the classification performances are evaluated based on 10-fold cross validation. We report the performance results from Jin et al. (2020) for all other baseline methods. In addition to the graph datasets used in their work, we include additional datasets; for these latter (last three columns in Table 4) we ran their code again to obtain results for SGC and MGC.

##### 4.2.2.3. Results

Table 5 shows the performance on the graph classification task with all the graph coarsening methods. For our method, the hyperparameters of the memory learning are tuned to minimize the loss of unsupervised task for each dataset. We can see that our method is competitive compared with all the other baseline methods across all the datasets. Our method has the best performance on 5 out of 8 datasets and a close second best on ENZYMES and TUMBLR. The drop in accuracy between the original graph and the coarsened graph is small (and sometimes the coarsened graph results in even higher accuracy).

**Table 5:** Average graph classification accuracy for the coarsened graph.

**Dataset**	**EM**	**LV**	**METIS**	**SC**	**SGC**	**MGC**	**HARP**	**GCN**	**Our**	**Original graph**
MUTAG	78.9	79.01	77.62	80.37	80.34	81.53	81.34	71.42	**83.50**	86.58
Enzymes	18.92	24.68	24.79	24.40	29.19	**30.89**	28.67	23.87	29.92	37.32
NCI109	61.35	60.49	61.64	62.57	63.69	**63.55**	62.72	56.42	59.42	64.93
Proteins	63.72	62.72	63.70	64.08	64.70	65.26	63.24	62.17	**66.07**	66.60
PTC_MR	48.56	50.24	49.34	50.16	52.76	52.28	52.56	51.86	**53.36**	53.72
TUMBLR	N/A	N/A	N/A	N/A	49.05	47.45	**49.57**	48.72	49.34	47.82
OHSU	N/A	N/A	N/A	N/A	58.03	58.03	59.42	60.71	**61.25**	55.89
REDDIT-BINARY	N/A	N/A	N/A	N/A	(>24 h)	(>24 h)	68.56	67.72	**69.83**	78.42

Bold value stands for the best performance for each dataset. N/A stands for results that are not reported in the prior literature.

#### 4.2.3. Block structure identification

In this section we use the stochastic block model of Holland et al. (1983) to generate synthetic graphs with some group structure, and then use different graph coarsening methods to simplify the graph. We test whether or not the graph coarsening methods can identify the group structure information, i.e., the nodes within the same group in the original graph ideally should be contracted as one single node in the coarsened graph. We adopt this setting to directly compare with previous work (Jin et al., 2020).

##### 4.2.3.1. Stochastic block model

The stochastic block model is a generative model for random graphs. The model tends to generate graphs containing communities; subsets of nodes characterized by being connected with one another with particular edge densities. For example, edges may be more common within communities than between communities. Suppose we have *n* communities, the model is parameterized as *A*∈[0, 1]^*n*×*n*^, where *A*[*i, j*] refers to the probability of connection between community *i* and *j*. The matrix *A* is symmetric (i.e., *A*[*i, j*] = *A*[*j, i*]) and *A*[*i, i*] refers to the probability of connection within the community.

The community is grouped based on clustering structure if *A*[*i, i*]>*A*[*i, j*] whenever *i*≠*j*, which is also referred as the assortative case. On the other hand, the community is grouped based on anti-clustering structure if *A*[*i, i*] < *A*[*i, j*] whenever *i*≠*j*, which is also referred as disassortative case. Our algorithm iteratively contracts the most similar node pairs, which identifies the clustering structure for the graph, and does the coarsening based on the clustering structure. Our algorithm works only in the assortative case where community is formed by clustering structure, thus we only focus on the assortative case in the stochastic block model.

##### 4.2.3.2. Baseline methods and metrics

We directly compare with SGC and MGC (Jin et al., 2020), which are considered to be the state-of-the-art coursening methods. We study the model performance for large graphs with thousands of nodes. We use the assortative stochastic block model to generate graphs with different sizes and different diagonal/off-diagonal probabilities for comparison. We evaluate the different graph coarsening methods by measuring the Normalized Mutual Information (NMI) (Estévez et al., 2009) between the block suggested by the model and the ground truth partition, since NMI is a common metric to check the partition discrepancy. The Mutual Information (MI) between two partitions *U* and *V* is defined as follow:


$$MI\left(U,V\right)=\overset{\left|U\right|}{\underset{i=1}{\sum }}\overset{\left|V\right|}{\underset{j=1}{\sum }}\frac{\left|U_{i}\cap V_{j}\right|}{N}\log\frac{N|U_{i}\cap V_{j}|}{\left|U_{i}||V_{j}\right|}$$

where |*U*_*i*_| is the number of nodes in partition *U*_*i*_, |*V*_*j*_| is the number of nodes in partition *V*_*j*_, *N* is the total number of nodes. NMI is a normalization of the MI score to scale the results between 0 and 1. For our model, we keep track the two nodes that are merged, and in this way we can get the group partition when the coarsening is finished.

##### 4.2.3.3. Results

Table 6 shows the performance of all the coarsening methods with different graph sizes and parameters. Here, we use *p* and *q* to denote the diagonal and off-diagonal probabilities in the stochastic block model, *N* and *n* to denote the number of nodes in the whole graph and the number of communities or groups. The results are averaged NMI scores for 10 runs.

**Table 6:** Average normalized mutual information for block recovery with different methods and the ground truth.

***N*, *n***	***p*, *q***	**MGC**	**SGC**	**Our**
*N* = 2, 000, *n* = 100	0.4, 0.01	0.47 (~7 h)	0.89 (~500 s)	**0.99 (** **~** **26 s)**
	0.5, 0.1	0.10 (~7 h)	0.39 (~500 s)	**0.40 (** **~** **26 s)**
	0.8, 0.3	0.09 (~7 h)	0.34 (~500 s)	**0.37 (** **~** **26 s)**
*N* = 4, 000, *n* = 200	0.4, 0.01	0.26 (~70 h)	0.90 (~3,700 s)	**0.97 (** **~** **120 s)**
	0.5, 0.1	0.09 (~70 h)	0.41 (~3,700 s)	**0.44 (** **~** **120 s)**
	0.8, 0.3	0.09 (~70 h)	0.40 (~3,700 s)	**0.44 (** **~** **120 s)**
*N* = 6, 000, *n* = 300	0.4, 0.01	N/A	0.87 (~12,000 s)	**0.88 (** **~** **300 s)**
	0.5, 0.1	N/A	0.43 (~12,000 s)	**0.48 (** **~** **300 s)**
	0.8, 0.3	N/A	0.40 (~12,000 s)	**0.47 (** **~** **300 s)**

The number in parenthesis shows the average running time per run. The last case for MGC is omitted (noted as N/A) due to the running time limit (we set it as 100 h). Bold value stands for the best performance for each dataset.

The ratio between *p* and *q* can be regarded as the significance indicator of the clustering structure in the graph. When the ratio is very large, it means the connections within the group are much more likely than inter-group connections. When this ratio decreases the distinction between within- vs. inter- become more subtle. Our method is more successful at identifying the group clustering structure, and it clearly outperforms the conventional methods in terms of the NMI score. On all the datasets, ranging from 2,000 to 6,000 nodes, our method yields the best results. Besides that, our algorithm is extremely fast compared with the other methods. It is around 20–40 times faster than the commonly used methods.

## 5. Conclusions

In this work we have proposed a framework for learning node embeddings using Modern Hopfield Networks in combination with the masked node training. The context of each node activates a set of memory vectors that are used for predicting the identity of the masked node. From the theoretical perspective, our main contribution is the extension of the results of Krotov and Hopfield (2016, 2020), Ramsauer et al. (2020), and Krotov (2021) to the setting where each data point (a given node in the network embedding problem) is represented by several distinct kinds of attributes (e.g., context node identity, target node identity, labels, etc.). Some of these attributes (e.g., masked node identity) can evolve in time using the Hopfield dynamics, while others (e.g., context) can be kept clamped to guide the dynamical trajectory in the direction of the appropriate (for that context) memory. The core computational strategy of Associative Memory naturally informs the appropriate pattern completion for the masked node and learns useful representations for the memory vectors, which can be utilized for multiple downstream tasks.

We have obtained strong results evaluating our framework on the node classification and link prediction tasks. We have also developed an Associative Memory based framework for graph coarsening. The coarsened graphs obtained by our method demonstrate excellent properties retaining valuable information from the original graphs. This desirable property of our method has been established on both natural and synthetic datasets. Our work opens up several avenues for future work. Our current work is based on purely structural node embeddings. It is of interest to extend our methods to consider content (e.g., taking into account both node and edge features) in addition to the structural context. Other directions include developing Hierarchical Associative Memories to capture higher-level graph features.

## Author's note

Recently, Associative Memory has attracted lots of attention due to the development of the Modern Hopfield Network. In this work, we design an Associative Memory update rule and its corresponding energy function suitable for the network embedding task. To our knowledge, it's the first attempt to apply Modern Hopfield Network to this fundamental graph learning task. We empirically show that the performance of our MHN-based embedding for the node classification, link prediction and graph coarsening downstream tasks is competitive with the commonly used matrix factorization methods and deep learning approaches.

## Data availability statement

The original contributions presented in the study are included in the article/Supplementary material, further inquiries can be directed to the corresponding author/s.

## Author contributions

All authors listed have made a substantial, direct, and intellectual contribution to the work and approved it for publication.

## Funding

This work was supported by the Rensselaer-IBM AI Research Collaboration, part of the IBM AI Horizons Network.

## Conflict of interest

Author DK was employed by IBM. YL was supported as an AI Horizons Network Scholar. The remaining author declares that the research was conducted in the absence of any commercial or financial relationships that could be construed as a potential conflict of interest.

## Publisher's note

All claims expressed in this article are solely those of the authors and do not necessarily represent those of their affiliated organizations, or those of the publisher, the editors and the reviewers. Any product that may be evaluated in this article, or claim that may be made by its manufacturer, is not guaranteed or endorsed by the publisher.
