# Supplementary material for: Modern Hopfield Networks for graph embedding
Source: Front Big Data. 2022 Nov 17;5:1044709. doi: 10.3389/fdata.2022.1044709 (PMC9713410; doi:10.3389/fdata.2022.1044709)
Supplement: Supplementary file 1 [file Presentation_1.pdf]

Supplementary Material

APPENDIX A. ENERGY FUNCTION OF THE MODERN HOPFIELD NETWORK FOR GRAPH EMBEDDING

In (Krotov and Hopfield, 2020) a continuous state and time Modern Hopfield Network was defined with the following dynamical equations:

$$\begin{cases} \tau_f \frac{dv_i}{dt} = \sum_{\mu=1}^{K} \xi_{i\mu} f_\mu - v_i + I_i \\ \tau_h \frac{dh_\mu}{dt} = \sum_{i=1}^{m} \xi_{\mu i} g_i - h_\mu \end{cases} \tag{S1}$$

In these equations, there are two types of neurons: feature neurons v_i and hidden neurons h_μ. The strength of the synaptic connections (weights) from v_i to h_μ is $\xi_{\mu i}$, and the strength of the synaptic connections from h_μ to v_i is $\xi_{i\mu}$. These two sets of weights are equal. The output of the memory neurons and the feature neurons are defined as f_μ and g_i respectively, which are nonlinear functions of the corresponding internal states of the neurons (which biophysically correspond to currents). The input I_i denotes the input current into the feature neurons. The time constants for the two groups of neurons are denoted by τ_f and τ_h. In this Appendix we show that our network can be reduced to this general Modern Hopfield Network (with some parameter selection as a special case). If this is the case, then we can use the energy function of (Krotov and Hopfield, 2020) for our MHN for graph setting.

In (Krotov and Hopfield, 2020), the activation functions f_μ and g_i are defined as the derivatives of the Lagrangian functions of the corresponding neurons.

$$f_\mu = \frac{\partial L_h}{\partial h_\mu} \quad \text{and} \quad g_i = \frac{\partial L_v}{\partial v_i}$$

In our model, both the input context vector v_{context} and the context memory block Ψ_{context} are unchanged during the retrieval process, i.e., their dot product is a constant K-dimensional vector $\epsilon_\mu = (\epsilon_1, \epsilon_2, ..., \epsilon_K)$. If we further set $\tau_h = 0$, $I_i = 0$, $\xi_{i\mu} = \xi_{\mu i} = (\Phi_{\text{target}})_{\mu i}$, $L_h = \log(\sum_{\mu=1}^{K} e^{(h_\mu + \epsilon_\mu)})$, $v_i = (v_{\text{target}})_i$ and $L_v = \frac{1}{2}\sum_i^m v_i^2$, the second equation in Eq. S1 can be solved at the fix point where

$$h_\mu = \sum_{i=1}^{m} (\Phi_{\text{target}})_{\mu i} v_i$$

Then if we substitute it into the first equation in Eq. S1, we have

$$\tau_f \frac{dv_i}{dt} = \sum_{\mu=1}^{K} (\Phi_{\text{target}})_{\mu i} f\Big(\sum_{j=1}^{m} (\Phi_{\text{target}})_{\mu j} v_j + \epsilon_\mu\Big) - v_i \tag{S2}$$

where f is the softmax function. This continuous time equation can be written in terms of finite differences

$$v_i^{(t+1)} = v_i^{(t)} + \frac{dt}{\tau_f}\Big[\sum_{\mu=1}^{K}(\Phi_{\text{target}})_{\mu i} f\Big(\sum_{j=1}^{m}(\Phi_{\text{target}})_{\mu j} v_j^{(t)} + \epsilon_\mu\Big) - v_i^{(t)}\Big] \tag{S3}$$

If we further define $\frac{dt}{\tau_f} = \alpha$ as the update rate, then that is exactly the update function of our model.

Note that both Lagrangian functions $L_h = \log(\sum_{\mu=1}^{K} e^{(h_\mu+\epsilon_\mu)})$ and $L_v = \frac{1}{2}\sum_i^m v_i^2$ are convex functions, i.e., their Hessian matrices are positive semi-definite. Following the derivation of (Krotov and Hopfield, 2020), it can be shown that the network dynamics minimize the following energy function

$$E = \frac{1}{2}\sum_{i=1}^{m}(v_{target})_i^2 - \log\Big[\sum_{\mu=1}^{K}\exp\Big(\sum_{i=1}^{m}(\Phi_{\text{target}})_{\mu i}(v_{target})_i + \epsilon_\mu\Big)\Big]$$

APPENDIX B. ABLATION STUDY FOR THE MODEL HOPFIELD NETWORKS WITH RESPECT TO THE NUMBER OF MEMORIES

In Table S1 we report the ablation study for our Hopfield Network with respect to different number of memories on all the three datasets. The table shows the average micro- and macro-f1 scores for node classification, and the AUC score for link prediction. We can observe that the performance of our model is fairly robust to the change in the number of memories, with relatively little variation, which can be considered a strength of our model.

		Node Classification		Link Prediction
Dataset	K	micro-f1	macro-f1	AUC
BlogCatalog	2000	40.26 (0.53)	24.23 (0.97)	0.92
	1600	40.52 (0.47)	24.43 (1.23)	0.89
	800	39.87 (0.66)	24.11 (0.87)	0.91
	400	38.76 (0.81)	23.87 (1.13)	0.87
	200	40.14 (0.37)	24.02 (0.86)	0.87
	100	38.64 (0.65)	23.65 (0.74)	0.85
PPI	2000	26.11 (1.48)	21.29 (1.36)	0.91
	1600	25.87 (0.98)	20.79 (1.03)	0.92
	800	26.02 (0.76)	21.17 (0.87)	0.91
	400	26.34 (0.81)	21.52 (1.23)	0.89
	200	26.22 (1.21)	21.23 (0.86)	0.90
	100	25.62 (1.32)	20.67 (0.83)	0.87
Wikipedia	2000	57.62 (0.98)	13.84 (0.73)	0.87
	1600	57.01 (0.74)	13.21 (0.65)	0.85
	800	56.98 (0.69)	12.94 (0.53)	0.86
	400	57.86 (0.82)	14.02 (1.33)	0.88
	200	57.98 (0.67)	13.92 (0.76)	0.84
	100	56.73 (0.75)	12.65 (0.57)	0.85

Table S1. Performance variations for node classification and link prediction tasks with respect to different memories. The number in the bracket denotes standard deviation.

APPENDIX C. PERFORMANCE ANALYSIS FOR GRAPH COARSENING

We observed that our model outperforms the baselines on the graph classification task for all datasets, except for NCI109. To further understand why our model does not perform so well on NCI109, we studied its network characteristics vis-a-vis the ENZYME dataset, where our model is the second best, and also PROTEINS and OHSU, where our model clearly outperforms the baselines.

Figure S1 shows the degree distribution for these four datasets. What we observe is that NCI109 has very sparse graphs (with max degree 5), whereas ENZYME is also on the sparser side, but with higher max and mean degrees. On the other hand PROTEINS and OHSU are more dense graphs with nodes having larger degrees. Given that our model is designed to learn prototypes, if nodes are very sparsely connected, it becomes harder to learn effective memories, and this is one of the reasons why we do not perform so well on NCI109.

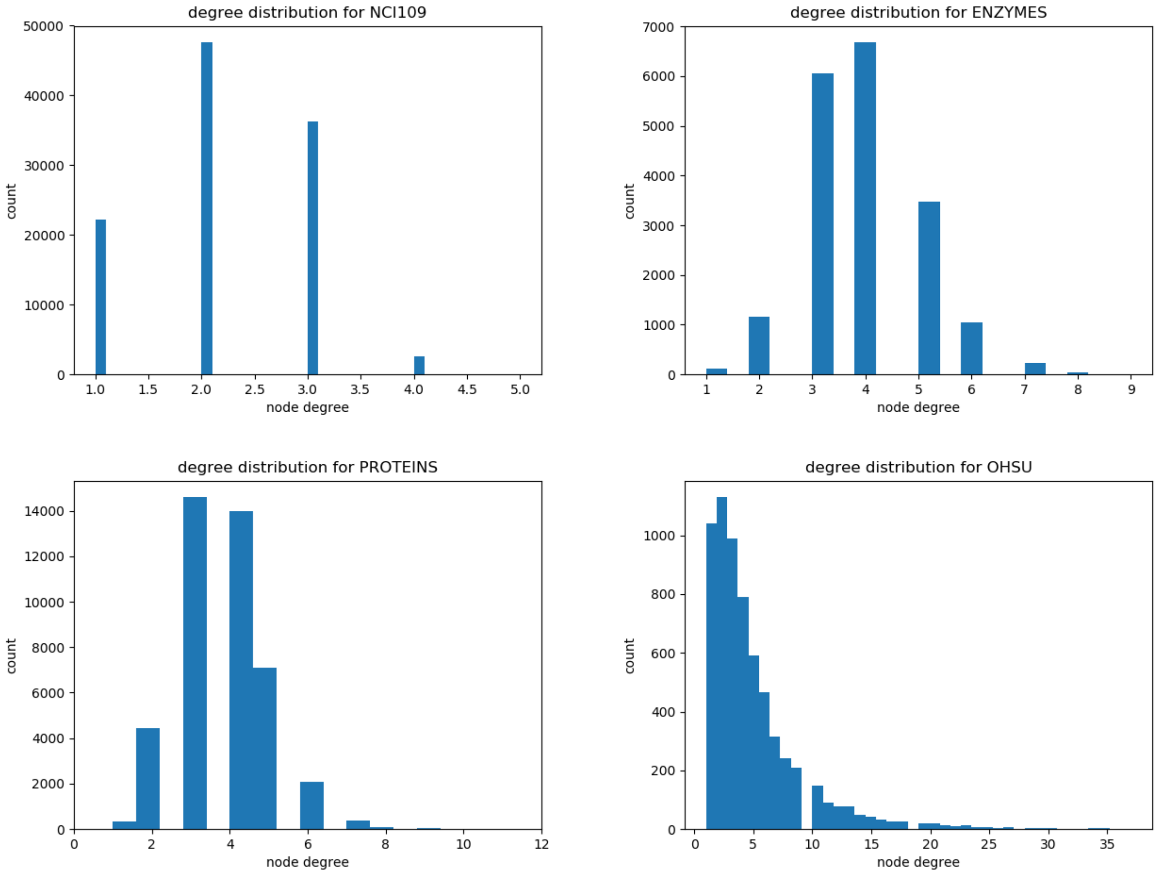

Figure S1. The degree distribution for NCI109, ENZYMES, PROTEINS and OHSU dataset. For each dataset, we count all the node degrees across the different graphs in the dataset.

REFERENCES

Krotov, D. and Hopfield, J. (2020). Large associative memory problem in neurobiology and machine learning. *arXiv preprint arXiv:2008.06996*
